# Thulium fiber laser vs. holmium laser enucleation of the prostate: results of a prospective randomized non-inferiority trial

**DOI:** 10.1007/s00345-023-04748-7

**Published:** 2024-01-20

**Authors:** Marina Kosiba, Maximilian Filzmayer, Maria N. Welte, Leonie Hügenell, Anna C. Keller, Miriam I. Traumann, Matthias J. Müller, Luis A. Kluth, Philipp C. Mandel, Felix K.-H. Chun, Andreas Becker

**Affiliations:** 1 Department of Urology, Goethe University Frankfurt, University Hospital, Frankfurt, Germany; 2 Urological Center at Boxberg, Neunkirchen, Germany

**Keywords:** Lower urinary tract symptoms, Laser enucleation of the prostate, Thulium fiber laser, Soltive™, Holmium laser, ThuFLEP, HoLEP

## Abstract

**Purpose:**

Holmium laser enucleation of the prostate (HoLEP) represents the current standard procedure for size-independent surgical therapy of benign prostatic obstruction (BPO). With advent of the novel laser technology thulium fiber laser (TFL), we hypothesized that the functional outcome of TFL enucleation of the prostate (ThuFLEP) is non-inferior compared to HoLEP.

**Methods:**

From October 2021 to October 2022, 150 patients with BPO were recruited for the prospective randomized trial in accordance with CONSORT. Stratified randomization into the arms ThuFLEP (*n* = 74) or HoLEP (*n* = 76) was carried out. The primary endpoint was non-inferior international prostate symptom score (IPSS) and quality of life (QoL) at three months after treatment. Secondary endpoints were rates of complications, peak flow, residual urine and operation times.

**Results:**

Preoperative characteristics showed no significant differences. Overall IPSS and QoL improved from 21 to 8 and 4 to 1.5, respectively, after three months of follow-up. No statistically significant differences between ThuFLEP and HoLEP were observed regarding median postoperative IPSS (8.5 vs. 7, *p* > 0.9), QoL (1 vs. 2, *p* = 0.6), residual urine (48 vs. 30ml, *p* = 0.065) and peak flow (19 vs. 17ml/s, *p* > 0.9). Similarly, safety profile was comparable with no statistically significant differences regarding rate of major complications (5.3 vs. 5.4%, *p* = 0.5), laser hemostasis time (3 vs. 2min, *p* = 0.2), use of additive electric coagulation (74 vs. 87%, *p* = 0.06) or electric coagulation time (8 vs. 8min, *p* = 0.4).

**Conclusions:**

In this prospective, randomized trial ThuFLEP showed non-inferior results compared to HoLEP in terms of functional outcomes measured by IPSS and QoL as primary endpoint.

**Trial registration number:**

DRKS00032699 (18.09.2023, retrospectively registered).

**Supplementary Information:**

The online version contains supplementary material available at 10.1007/s00345-023-04748-7.

## Introduction

Holmium laser enucleation of the prostate (HoLEP) has proven to be a safe and effective minimally invasive surgical treatment for bladder outlet obstruction (BOO) due to benign prostatic hyperplasia (BPH) [1, 2]. It can be performed regardless of size and with less morbidity as compared to transurethral resection of the prostate (TURP) and open simple prostatectomy, even in patients requiring anticoagulation [3–5].

With the thulium fiber laser (TFL), a novel energy source with different physical properties is available, which may have a positive impact on the safety profile of the LEP [6–10]. The wavelength of the TFL (1940nm) has a beneficial energy absorption maximum in water, which leads to a more shallow tissue penetration depth and reduced carbonization [11–15]. The TFL allows a wide variety of laser settings [11]. The potentially harmful effects are described as reduced while the hemostatic effect appears to be improved [11, 14, 16]. Due to less vaporization of the water between laser fiber and tissue TFL creates less bursting energy for tissue dissection and therefore has an improved cutting efficiency [11, 15].

In the studies available to date, the TFL enucleation of the prostate (ThuFLEP) has been shown to be equivalent to the HoLEP in terms of absolute operating time, enucleation and morcellation speed and functional results [10, 17–20]. However, most of the published evidence is of retrospective nature or based on small number of cases [8]. The only randomized prospective study, which was conducted by Enikeev et al. defined the severity of urinary incontinence according to the ICIQ-MLUTS as primary outcome [10]. There is still limited evidence on the use and clinical outcomes of ThuFLEP [8, 21, 22]. To fill this void, we conducted the following prospective randomized trial. Our goal is to verify that ThuFLEP is not inferior to HoLEP regarding functional outcomes, measured by IPSS and QoL three months after surgery. Urinary continence, objective voiding parameters, safety profile and efficiency represented secondary outcomes.

## Material and methods

### Data collection

This prospective randomized non-inferiority trial was performed at the University Hospital in Frankfurt after obtaining approval from the Institutional Ethics Committee (2021–171, approval No. E 98/21). We included all patients scheduled for LEP. Exclusion criteria were prostate volume measured by transrectal ultrasound (TRUS) of less than 30ccm, history of urethral stricture, preoperative evidence of prostate carcinoma or confirmed neurogenic bladder emptying disorder. From October 2021 to October 2022, 268 patients were planned for LEP, of whom 158 patients gave informed written consent.

Following strata were used for a stratified randomization into the two arms ThuFLEP and HoLEP: indwelling transurethral catheter vs. IPSS ≥ 20 vs. IPSS < 20, age < 70 years vs. age ≥ 70 years and prostate volume < 80ccm vs. ≥ 80ccm.

For ThuFLEP a Soltive™ SuperPulsed TFL (Olympus) with 550nm laser fiber (Olympus), 1.5J pulse energy and 40 Hz frequency setting was used. For HoLEP a high-power holmium laser (MOSES™ Pulse 120H, Boston Scientific) with 550nm laser fiber (Slim Line, Boston Scientific), 1.4J pulse energy and 50 Hz frequency setting was used. MOSES™ pulse modulation was deactivated. Enucleation efficiency was defined as enucleation weight per enucleation time. Postoperative complications were recorded according to the Clavien-Dindo (CLD) classification system [23]. Five surgeons (2 high-volume surgeons: mean experience 592 cases, 3 low-volume surgeons: mean experience 57 cases) were assigned randomly to both treatment groups according to personal availabilities.

PROMs (patient reported outcome measures) were collected preoperatively and three months postoperatively including standardized validated questionnaires: international prostate symptom score (IPSS), quality of life (QoL) and international consultation of incontinence questionnaire short form (ICIQ-SF). Pre- and postoperative continence was defined as ICIQ-SF ≤ 4 or usage of at most one security pad per day.

### Statistical analysis

Based on an assumed standard deviation regarding IPSS reduction at three months of 4 points, a non-inferiority margin of 2 points (half the standard deviation) was determined for the sample size calculation. Totally 128 patients should be included for the analysis of the study for an 80% statistical power with the upper limit of a one-sided 95% confidence interval exceeding a > 5% difference in favor of the standard treatment group. Considering a drop-off rate of 15%, 152 study participants should be randomized per minimum.

Multivariable regression models tested the effect of the laser technology on the target criterions mentioned above. In all statistical analyses, R software environment for statistical computing (version 3.6.1) was used. The level of significance was set at *p* < 0.05.

## Results

158 patients consented to be enrolled in the study, in one case an intraoperative urethral stricture was found, four patients were lost to follow-up and in three cases a randomization error occurred. These eight patients were excluded from data analysis.

### Descriptive characteristics of the study population

**Fig. 1:**
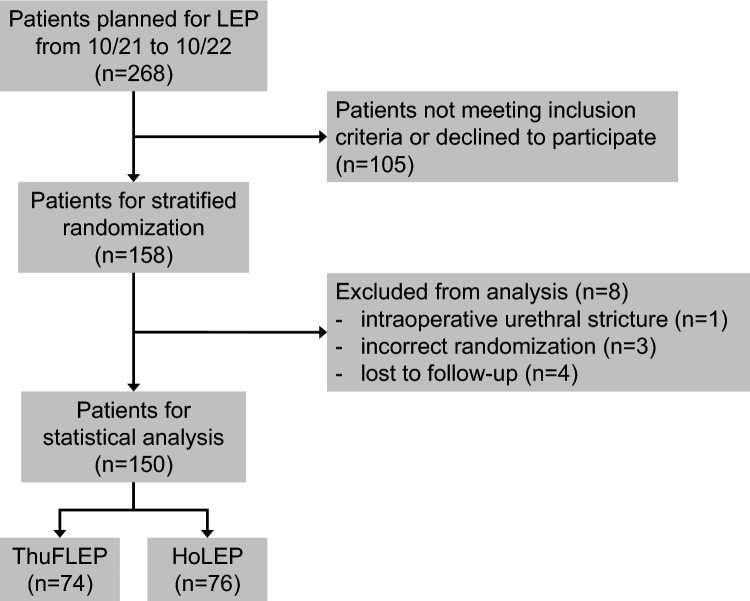
CONSORT flow diagram depicting the patient inclusion path. Based on an estimated drop-off rate of 15% and a final drop-off rate of only 5% we exceeded the initial sample size calculation of at least 128 patients by including 150 randomized patients to the statistical analysis

Finally, data from 150 patients could be included for the analysis, so that the initial sample size calculation of a minimum of 128 patients was met (Fig. 1). 74 (49%) received ThuFLEP and 76 (51%) received HoLEP. The mean age was 69 years. The mean prostate volume was 80ccm. All preoperative characteristics did not show significant differences (*p* > 0.05, Table 1).

**Table 1:** Pre, peri- and postoperative characteristics of 150 patients randomized to Thulium Fiber Laser Enucleation (ThuFLEP) or Holmium Laser Enucleation (HoLEP) at the University Hospital Frankfurt in the period from 10/21 to 10/22

	N	Overall^1^	ThuFLEP *N* = 74 (49%)^1^	HoLEP *N* = 76 (51%)^1^	*p* value^2^
Age [years]	150	69 (65, 74)	69 (65, 74)	69 (65, 74)	0.6
Preoperative PSA [ng/ml]	134	4.7 (2.8, 7.9)	4.9 (2.8, 7.1)	4.1 (2.9, 8.7)	0.6
Prostate volume [ccm]	150	80 (63, 100)	76 (62, 95)	80 (65, 107)	0.3
ASA risk score	150				> 0.9
I/II		108 (72%)	53 (72%)	55 (72%)	
III/IV		42 (28%)	21 (28%)	21 (28%)	
Preoperative IPSS	115	21 (16, 26)	22 (17, 26)	20 (16, 25)	0.5
IPSS at three months after surgery	129	8.0 (3.0, 12.0)	8.5 (3.0, 12.0)	7.0 (4.0, 12.0)	> 0.9
Preoperative QoL	131	4.00 (3.50, 5.00)	4.50 (3.75, 5.00)	4.00 (3.50, 5.00)	0.6
QoL at three months after surgery	128	1.50 (1.00, 3.00)	1.00 (1.00, 3.00)	2.00 (1.00, 3.00)	0.6
Preoperative Qmax [ml/s]	98	9.3 (6.7, 13.0)	9.4 (6.8, 13.4)	9.2 (6.7, 11.9)	0.5
Qmax at discharge [ml/s]	102	18 (11, 27)	19 (10, 26)	17 (12, 27)	> 0.9
Preoperative PVR [ml]	107	100 (40, 200)	100 (50, 150)	100 (40, 200)	0.9
PVR at discharge [ml]	132	39 (20, 60)	48 (26, 68)	30 (20, 58)	0.07
Preoperative indwelling catheter	140				0.7
No		106 (76%)	52 (74%)	54 (77%)	
Yes		34 (24%)	18 (26%)	16 (23%)	
Indwelling catheter at three months after surgery	149				0.6
No		146 (98%)	72 (97%)	74 (99%)	
Yes		3 (2.0%)	2 (2.7%)	1 (1.3%)	
Preoperative ICIQ-SF	119	5.0 (0.0, 11.0)	4.0 (0.0, 12.0)	6.0 (0.0, 10.0)	0.7
ICIQ-SF at three months after surgery	128	4.0 (0.0, 9.0)	4.0 (0.0, 9.0)	4.0 (0.0, 8.5)	> 0.9
Preoperative continence^3^	123				0.4
No		32 (26%)	14 (23%)	18 (29%)	
Yes		91 (74%)	47 (77%)	44 (71%)	
Continence at three months after surgery^3^	128				0.3
No		23 (18%)	13 (21%)	10 (15%)	
Yes		105 (82%)	48 (79%)	57 (85%)	
High-volume surgeon^4^	150				0.8
No		65 (43%)	33 (45%)	32 (42%)	
Yes		85 (57%)	41 (55%)	44 (58%)	
Enucleation weight [g]	150	52 (36, 70)	50 (34, 70)	55 (39, 70)	0.4
Overall operation time [min]	150	58 (45, 78)	58 (45, 84)	56 (43, 75)	0.12
Enucleation time [min]	126	30 (22, 45)	36 (26, 45)	27 (21, 43)	**0.016**
Enucleation efficiency [g/min]	126	1.62 (1.05, 2.40)	1.45 (0.91, 2.00)	2.03 (1.17, 2.96)	**0.001**
Laser time [min]	117	22 (17, 29)	24 (17, 32)	20 (17, 28)	0.10
Laser hemostasis time [min]	113	3.00 (1.00, 4.00)	2.00 (1.00, 4.00)	3.00 (2.00, 5.00)	0.2
Electric coagulation	135				0.06
No		27 (20%)	9 (13%)	18 (26%)	
Yes		108 (80%)	58 (87%)	50 (74%)	
Electric coagulation time [min]	106	8 (5, 14)	8 (6, 15)	8 (5, 11)	0.4
Morcellation time [min]	120	7 (5, 13)	7 (4, 13)	8 (6, 14)	0.13
Complications	150				0.5
No (CLD 0)		125 (83%)	59 (80%)	66 (87%)	
Minor (CLD I-IIIa)		17 (11%)	11 (15%)	6 (7.9%)	
Major (CLD IIIb)^5^		8 (5.3%)	4 (5.4%)	4 (5.3%)	

^1^Mean/median (IQR); n (%)

^2^Wilcoxon rank sum test; Pearson's Chi-square test; Fisher's exact test

^3^Defined as ICIQ-SF ≤ 4 or at most one security pad

^4^Defined as surgeon with caseload over 400 cases

^5^No CLD IV-V complications occurred

### Functional outcomes

The three-month follow-up showed an improvement of overall IPSS and QoL from 21 preoperatively to 8 and from 4 to 1.5 points. No statistically significant differences between ThuFLEP and HoLEP were observed regarding the primary endpoints median postoperative IPSS (8.5 vs. 7, *p* > 0.9) and QoL (1 vs. 2, *p* = 0.6). The preoperative mean peak flow (Qmax) of 9.3ml/s and post voiding residual urine (PVR) of 100 ml improved to 18ml/s and PVR of 39ml after surgery. No statistically significant differences regarding postoperative Qmax (19 vs. 17ml/s, *p* > 0.9) and PVR (48 vs. 30ml, *p* = 0.065) were observed between the two treatment groups. Regarding urinary incontinence, only 74% of the patients were continent preoperatively vs. 82% at 3 months after the operation and the mean ICIQ-SF was 5 vs. 4 points before and after the operation. Three months after surgery, 2% of the patients still were supplied with an indwelling catheter vs. 24% preoperatively. No statistically significant differences between ThuFLEP and HoLEP were observed regarding rates of continence (79 vs. 85%, *p* = 0.3), ICIQ-SF (4 vs. 4 points, *p* > 0.9) and rates of indwelling catheter (8.1 vs. 3.9%, *p* = 0.3).

### Perioperative efficacy and safety outcomes

Overall operation time (58 vs. 56min, *p* = 0.12) and morcellation time (7 vs. 8min, *p* = 0.13) showed no significant differences between ThuFLEP and HoLEP. However, HoLEP showed a significantly faster enucleation time of 27min and higher enucleation efficiency of 2.03g/min than ThuFLEP with 36min (*p* = 0.02) and 1.45g/min (*p* = 0.001). There were no significant differences regarding minor (15 vs. 7.9%, *p* = 0.5) or major (5.4 vs. 5.3%, *p* = 0.5) complication rates in ThuFLEP vs. HoLEP. Furthermore, there were no significant differences regarding laser hemostasis time (2 vs. 3min, *p* = 0.2), the use of additive electric coagulation (87 vs. 74%, *p* = 0.06) or electric coagulation time (8 vs. 8min, *p* = 0.4). Regarding possible learning curve effects using the novel laser source, we examined the second half of each surgeon's ThuFLEP cases as a subgroup comparison. HoLEP also showed a significantly faster enucleation time (27 vs. 37min, *p* = 0.04) and faster enucleation efficiency (2.03 vs. 1.13min/g, *p* < 0.001) compared to these last ThuFLEP cases (supplementary table).

### Multivariable regression models

Multivariable linear regression models were fitted to predict enucleation time according to the laser source adjusted for enucleation weight, age, surgeon’s caseload, ASA (American Society of Anesthesiologists) risk status and occurrence of complications. The use of the TFL resulted in a significant increase in enucleation time (OR 1.41, *p* < 0.001). Furthermore, also the surgeon’s caseload (OR 0.53, *p* < 0.001) and the enucleation weight (OR 1.01, *p* < 0.001) were significant predictors of prolonged enucleation time (Table 2). Multivariable logistic regression models were fitted to predict major complications according to the laser source adjusted for age, operation time and enucleation weight. The laser source was not a significant predictor of complications (supplementary table).

**Table 2:** Multivariable linear regression model predicting enucleation time according to the laser source adjusted for age, prostate enucleate weight, surgeon’s caseload, ASA status and occurrence of complications

	Odds Ratio	95% confidence interval		*p* value
		2.5%	97.5%	
ThuFLEP	1.41	1.24	1.60	** < 0.001**
Age [years]	1.00	0.99	1.01	0.7
Enucleation weight [g]	1.01	1.01	1.01	** < 0.001**
High-volume surgeon^1^	0.53	0.46	0.61	** < 0.001**
ASA risk classification III/IV	0.94	0.82	1.07	0.4
Minor complication (CLD I-IIIa)	1.07	0.92	1.25	0.4
Major complication (CLD IIIb)^2^	0.98	0.72	1.26	0.9

^1^Defined as surgeon with caseload over 400 cases

^2^No CLD IV-V complications occurred

## Discussion

There is still limited evidence on the use and clinical outcomes of ThuFLEP. To fill this void, we conducted the following prospective randomized trial and made several important observations.

First, our study’s primary outcome revealed no significant differences in functional outcomes measured by IPSS and QoL between HoLEP and ThuFLEP. Possible differences were suspected in theoretical tissue penetration depth, carbonization effects and heat development due to different energy absorption maxima of the two lasers in water tissue [11, 15]. However, in our study the subjective questionnaire scores and objective functional assessment did not differ between the two groups at 3 months of follow-up. This finding is in line with the exploratory analyses of the randomized prospective study by Enikeev et al. and the review of Pang et al. [10, 24].

Second, we found no differences in safety outcomes. Discussions about possible safety differences between TFL and holmium laser arose from the same reasons as mentioned above regarding functional outcomes [11]. The first ex vivo trials showed significant differences between the laser sources with improved hemostatic effect [11, 14, 16]. On the contrary, one ex vivo trial showed comparable coagulation between the two laser sources and another ex vivo trial even postulated a significant better coagulation with the holmium laser than with TFL [25, 26]. A systemic review of experimental studies postulated similar temperature changes caused by TFL and holmium laser [15]. Taken together, the ex vivo data are inhomogeneous. Our study observed no difference in all investigated hemostasis parameters, such as hemostasis time, additional use of electric coagulation for hemostasis or complications. This finding is supported by previous clinical studies investigating the safety of ThuFLEP [10, 17, 19, 24]. Only one publication comparing the TFL with holmium laser using MOSES™ 2.0 with BPH mode described significantly longer hemostasis time for TFL while the hemoglobin drop was not significantly different [20]. It is unclear to what extent the MOSES™ technology influenced this comparison [27].

Third, we found a higher enucleation efficiency in HoLEP cases compared to ThuFLEP cases in exploratory analyses. A possible explanation for this significant speed difference might be due to the laser characteristics. Tissue rupturing due to fast vaporization of the water between laser fiber and tissue as it is typical for the holmium laser may achieve better tissue separation than a sharp incision which is typical for the TFL [11]. In two ex vivo trials the tissue separation depth was greater with the holmium laser than TFL and objectively preferred by the surgeons [16, 25]. In three in vivo studies available to date, ThuFLEP was equivalent to HoLEP in terms of enucleation time and efficiency [10, 12, 18]. Only one trial described a better enucleation efficiency for HoLEP using MOSES™ 2.0 with BPH mode compared to ThuFLEP [20]. To what extent the MOSES™ technology could have influenced the comparison here is part of future research. However, our study showed no significant differences regarding overall operation time and morcellation time. This finding is supported by all previous data and the review of Taratkin et al. [10, 18, 19, 22]. Although the TFL was a relatively new laser source for our surgeons, who were all used to the holmium laser, our reported ThuFLEP enucleation efficiency of 1.45g/min is still higher than achieved in other publications (1.04 to 1.40g/min) [8, 10, 13, 17, 19, 20, 28]. This suggests that ThuFLEP has already been performed efficient and concerns about the learning curve can be put into perspective [12, 29].

Taken together, the results confirm that ThuFLEP is not inferior to HoLEP regarding functional outcomes, measured by IPSS and QoL 3 months after surgery.

### Limitations

This study was designed to provide best evidence regarding the non-inferiority of ThuFLEP vs. HoLEP. Nevertheless, also a prospective randomized trial is not devoid of limitations. The follow-up of the micturition symptoms was assessed without additional objective assessment of the voiding situation except at discharge. Thus, we cannot provide objective measurements such as Qmax and PVR for the follow-up time. However, the main target criterion of the study was to assess the subjective micturition performance and its related quality of life and there is evidence that the collection of PROMs can measure differences in subjective effects of health care interventions best [30]. All other analyses were just exploratory evaluations. Thus, conclusions drawn from our exploratory analysis regarding the operation times should bare this in mind. Electric coagulation was applied at the discretion of the surgeon at a relatively high rate, but it did not differ according to high- vs low-volume surgeons and was comparable within the two treatment groups. Moreover, the personal experience of each surgeon could influence the operation times and outcomes. However, we avoided assessing single surgeons’ outcomes and evaluated pooled results from five LEP surgeons, which allows to assess the operation technique as a whole. In addition, the proportion of experienced surgeons was distributed equally between the two treatment groups. Furthermore, late complications as bladder neck contracture and urethral stricture could not be evaluated as the current study focused on short-term functional outcomes and was not designed to estimate late complications. Finally, our prospective database may be influenced by a negative selection bias regarding the admittance of patients with a particularly high perioperative risk to our tertiary care university center. Nevertheless, the preoperative characteristics of our patients sampled a typical LEP collective [24]. Moreover, we included all consenting patients planned for LEP at our institution to allow most comprehensive analyses. This trial was conducted without professional study support and designed and carried out by clinical urologists.

## Conclusion

In this prospective, randomized study ThuFLEP is not inferior to HoLEP in terms of functional outcomes. In addition, a comparable safety profile and similar overall operation time was shown. Both ThuFLEP and HoLEP are effective and safe ways of treating benign prostatic obstruction.

## Supplementary Information

Below is the link to the electronic supplementary material.

Supplementary file1 (DOCX 27 KB)

## Data Availability

The data that support the findings of this study are available from the corresponding author upon reasonable request.
