# Supplementary material for: Thulium fiber laser vs. holmium laser enucleation of the prostate: results of a prospective randomized non-inferiority trial
Source: World J Urol. 2024 Jan 20;42(1):49. doi: 10.1007/s00345-023-04748-7 (PMC10799774; doi:10.1007/s00345-023-04748-7)
Supplement: Supplementary file 1 — Supplementary file1 (DOCX 27 KB) [file 345_2023_4748_MOESM1_ESM.docx]

**Table 2 (supplementary):** Multivariable linear regression model predicting major complications according to the laser source adjusted for age, prostate enucleate weight and operation time

|  | **Odds Ratio** | **95% Confidence Interval** | | **p-value** |
| --- | --- | --- | --- | --- |
|  |  | **2.5 %** | **97.5 %** |  |
| **ThuFLEP** | 1.09 | 0.24 | 4.93 | 0.9 |
| **Age [years]** | 1.05 | 0.95 | 1.17 | 0.3 |
| **Enucleation weight [g]** | 1.00 | 0.97 | 1.03 | 0.9 |
| **Overall operation time [min]** | 0.99 | 0.95 | 1.02 | 0.6 |

**Table 3 (supplementary):** Perioperative characteristics in the sensitivity analysis with only the second half of each surgeon’s ThuFLEP cases versus all HoLEP cases

|  | **N** | **Overall^1^** | **ThuFLEP**  **N=40 (34%)^1^** | **HoLEP**  **N=76 (66%)^1^** | **p-value^2^** |
| --- | --- | --- | --- | --- | --- |
| **Enucleation weight [g]** | 116 | 52 (35, 69) | 44 (30, 69) | 55 (39, 70) | 0.12 |
| **Overall operation time [min]** | 116 | 58 (44, 78) | 60 (45, 90) | 56 (43, 75) | 0.09 |
| **Enucleation time [min]** | 95 | 30 (22, 44) | 37 (24, 46) | 27 (21, 43) | **0.038** |
| **Enucleation efficiency [g/min]** | 95 | 1.63 (1.03, 2.49) | 1.13 (0.85, 1.77) | 2.03 (1.17, 2.96) | **<0.001** |
| **Laser time [min]** | 88 | 22 (17, 30) | 26 (17, 35) | 20 (17, 28) | 0.09 |
| **Laser hemostasis time [min]** | 88 | 3.00 (1.00, 5.00) | 3.00 (1.00, 4.50) | 3.00 (2.00, 5.00) | 0.4 |
| **Electric coagulation** | 102 |  |  |  | **0.014** |
| No |  | 20 (20%) | 2 (5.9%) | 18 (26%) |  |
| Yes |  | 82 (80%) | 32 (94%) | 50 (74%) |  |
| **Electric coagulation time [min]** | 79 | 8 (5, 13) | 8 (6, 14) | 8 (5, 11) | 0.6 |
| **Morcellation time [min]** | 92 | 8 (5, 14) | 7 (4, 13) | 8 (6, 14) | 0.3 |
| **Complications** | 116 |  |  |  | 0.8 |
| No (CLD 0) |  | 101 (87%) | 35 (88%) | 66 (87%) |  |
| Minor (CLD I-IIIa) |  | 7 (6.0%) | 3 (7.5%) | 4 (5.3%) |  |
| Major (CLD IIIb-V) |  | 8 (6.9%) | 2 (5.0%) | 6 (7.9%) |  |

^1^ Median (IQR); n (%)

^2^ Wilcoxon rank sum test; Pearson's Chi-square test; Fisher's exact test

Abbreviations:

SoLEP = Soltive™ Laser enculeation of the prostate

HoLEP = Holmium Laser enculeation of the prostate

CLD = Clavien-Dindo

IQR = interquartile ranges
